# Changes in spinopelvic sagittal parameters and functional outcomes after unilateral microscopic decompression for lumbar spinal stenosis

**DOI:** 10.3389/fmed.2026.1955497

**Published:** 2026-10-02

**Authors:** Keyu Zhao, Jiale Wang, Hao Liu, Xiaoyang Tao, Tiantian Wang, Junjie Kang, Wupeng Yang, Yonghua Wang

**Affiliations:** 1 Department of Orthopedics, Ordos Central Hospital, Ordos, Inner Mongolia Autonomous Region, China; 2 Ordos Clinical Medical College, Inner Mongolia Medical University, Ordos, China; 3 Department of Orthopedics, The First Hospital of Lanzhou University, Lanzhou, China

**Keywords:** correlation, lumbar lordosis, lumbar spinal stenosis, minimally invasive decompression, oswestry disability index, sagittal alignment

## Abstract

**Background:**

We evaluated short-term clinical and standing sagittal changes after unilateral microscopic channel-assisted decompression for lumbar spinal stenosis and examined their associations with disability and leg-pain improvement.

**Methods:**

This retrospective cohort included 68 patients treated between June 2024 and June 2025 and followed for 6–12 months. Oswestry Disability Index (ODI), leg-pain visual analog scale (VAS), pelvic tilt (PT), pelvic incidence (PI), sacral slope (SS), lumbar lordosis (LL), sagittal vertical axis (SVA), and thoracic kyphosis (TK) were assessed before surgery and at final follow-up. Changes were calculated as final follow-up minus preoperative values. Correlations with ODI and VAS improvement rates were tested with Holm correction. Interobserver and intraobserver reliability were assessed using intraclass correlation coefficients. An adjusted association analysis included age, body mass index, baseline ODI, and decompression extent.

**Results:**

ODI and VAS decreased significantly after surgery. PT and SVA decreased, whereas SS, LL, and TK increased; PI did not change significantly. The increase in LL was modestly associated with ODI improvement (Pearson r = 0.360, 95% CI 0.133–0.551; Holm-adjusted *p* = 0.026). No sagittal parameter was significantly correlated with VAS improvement after correction. In the adjusted analysis, *Δ*LL was not independently associated with ODI improvement (B = −0.108, 95% CI −0.276–0.060; *p* = 0.207). Follow-up duration was not associated with *Δ*LL or *Δ*SVA. Reliability was good to excellent.

**Conclusion:**

Clinical outcomes and standing sagittal parameters changed over follow-up. In this cohort, a greater increase in LL was modestly associated with greater ODI improvement only in the unadjusted analysis. The sagittal changes may represent functional postural adaptation rather than structural correction, but the observational design does not establish causality.

## Introduction

Lumbar spinal stenosis (LSS) is common in older adults and often causes neurogenic claudication, radicular leg pain, and functional limitation (1). When symptoms persist despite conservative treatment, surgical decompression can improve pain and disability (2). Minimally invasive decompression is an established surgical option (3). A unilateral microscopic approach permits bilateral decompression while preserving the contralateral lamina, spinous process, posterior ligamentous complex, and as much of the facet joint as possible (4). This approach is suited to patients without preoperative instability who do not require fusion.

Spinopelvic alignment helps maintain an energy-efficient standing posture (5), and spinal alignment changes reciprocally with pelvic compensation (6). Patients with symptomatic LSS often flex the trunk to reduce neural compression and improve walking tolerance (7). Changes in lumbar configuration and paraspinal musculature have also been described in this population (8). This pain-avoidant posture may reduce lumbar lordosis (LL), increase pelvic compensation, and alter global sagittal alignment (9). Unlike fixed deformity, some sagittal malalignment in LSS may be functional and reversible after neural decompression.

Decompression-only cohorts have reported postoperative increases in LL and reductions in compensatory sagittal parameters (10). At one year, Madkouri et al. observed increased LL and decreased SVA after isolated decompression (11), while a prospective cohort reported improvements in SVA and PI–LL mismatch (12). Similar changes have been described after microendoscopic decompression (13). The magnitude of change varies across studies, and its relationship with patient-reported outcomes remains uncertain (14). Because these operations do not include fusion or corrective instrumentation, postoperative radiographic changes may reflect recovery of standing posture rather than structural correction.

We evaluated clinical outcomes and standing sagittal parameters after unilateral microscopic channel-assisted decompression in patients with one- or two-level LSS and no dynamic instability. The primary question was whether changes in PT, SS, LL, SVA, and thoracic kyphosis (TK) were associated with improvement in ODI and leg-pain VAS. We expected the change in LL to show the strongest relationship with functional improvement.

## Materials and methods

### Study design and participants

This single-center retrospective cohort study included consecutive patients with LSS who underwent unilateral microscopic channel-assisted lumbar decompression in the Department of Spine Surgery, Ordos Central Hospital, between June 2024 and June 2025. The report was prepared in accordance with the Strengthening the Reporting of Observational Studies in Epidemiology (STROBE) recommendations (15).

Patients were eligible when symptoms and neurological findings were consistent with imaging-confirmed one- or two-level LSS, leg-pain VAS was greater than 4, and symptoms had persisted or progressed despite 3–6 months of conservative treatment. The number of decompressed levels was determined clinically rather than randomly: one level was treated when a single stenotic level was concordant with the patient's symptoms and neurological findings, whereas two levels were treated only when both stenotic levels were radiographically abnormal and clinically symptomatic. Of 96 operated patients screened, 28 were excluded because postoperative whole-spine radiographs were incomplete or loss to follow-up precluded retrieval of final VAS and/or ODI scores. Other exclusion criteria were lumbar infection, fracture, tumor, previous lumbar surgery, spondylolisthesis or deformity requiring fusion, severe hip or knee osteoarthritis interfering with standing assessment, and neurological disorders affecting gait. Patient selection is summarized in Figure 1.

**Figure 1:**
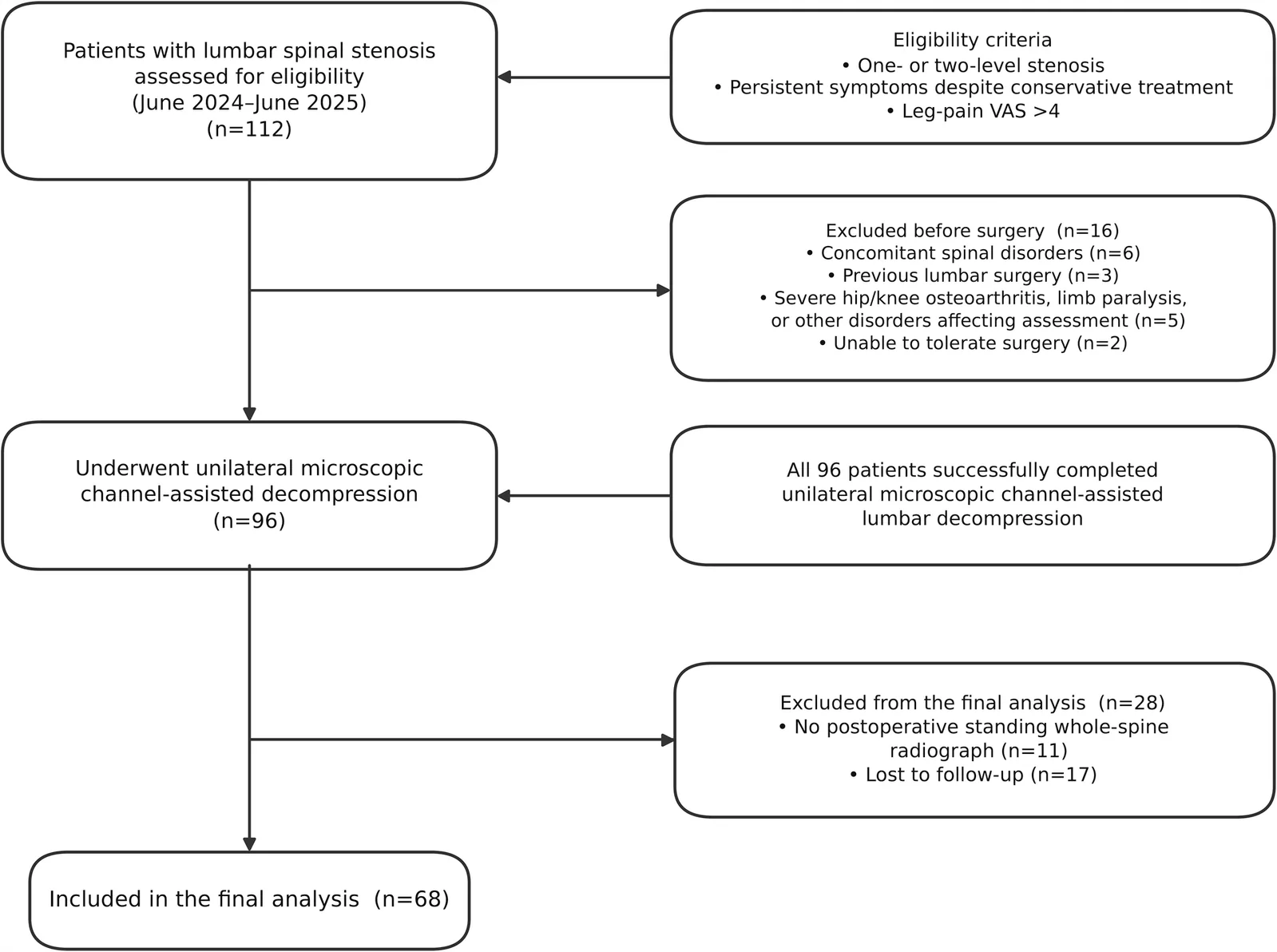
Flowchart of patient enrollment. VAS, visual analog scale. Alt text: Patient-enrollment flowchart showing screening, exclusions, and the final cohort of 68 patients.

Standing lumbar flexion-extension radiographs were obtained preoperatively in all patients specifically to assess dynamic stability. Because a universal threshold has not been established, we prespecified a sagittal-translation difference of ≥3 mm between flexion and extension as the operational definition of instability; this value lies within the 2–4 mm range most commonly selected by surveyed spine surgeons and matches a published definition of dynamic instability (16, 17). Patients meeting this criterion were not considered for decompression alone and were evaluated for fusion. All 68 included patients had translation differences <3 mm.

The protocol was approved by the Ethics Committee of Ordos Central Hospital, Inner Mongolia Medical University (approval No. 2022-012), and the study was conducted in accordance with the Declaration of Helsinki. Written informed consent for clinical assessment, imaging, and treatment was obtained from all participants.

### Surgical procedure

All procedures were performed by the same senior spine surgeon. Surgical levels were selected from concordant symptoms, neurological findings, and stenosis on MRI: one symptomatic level was decompressed in the single-level group, while both clinically concordant stenotic levels were decompressed in the double-level group. The symptomatic interlaminar space was localized under C-arm fluoroscopy, and a tubular working channel was introduced through a unilateral approach. Under microscopic visualization, a limited laminotomy was performed and the hypertrophic ligamentum flavum and compressive osseous structures were removed. Undercutting decompression was extended toward the contralateral side when required, while preserving the facet joint and posterior ligamentous complex. Decompression was considered adequate when the dural sac and affected nerve roots were freely mobile. No patient underwent fusion or corrective instrumentation. Representative operative images are shown in Figure 2.

**Figure 2:**
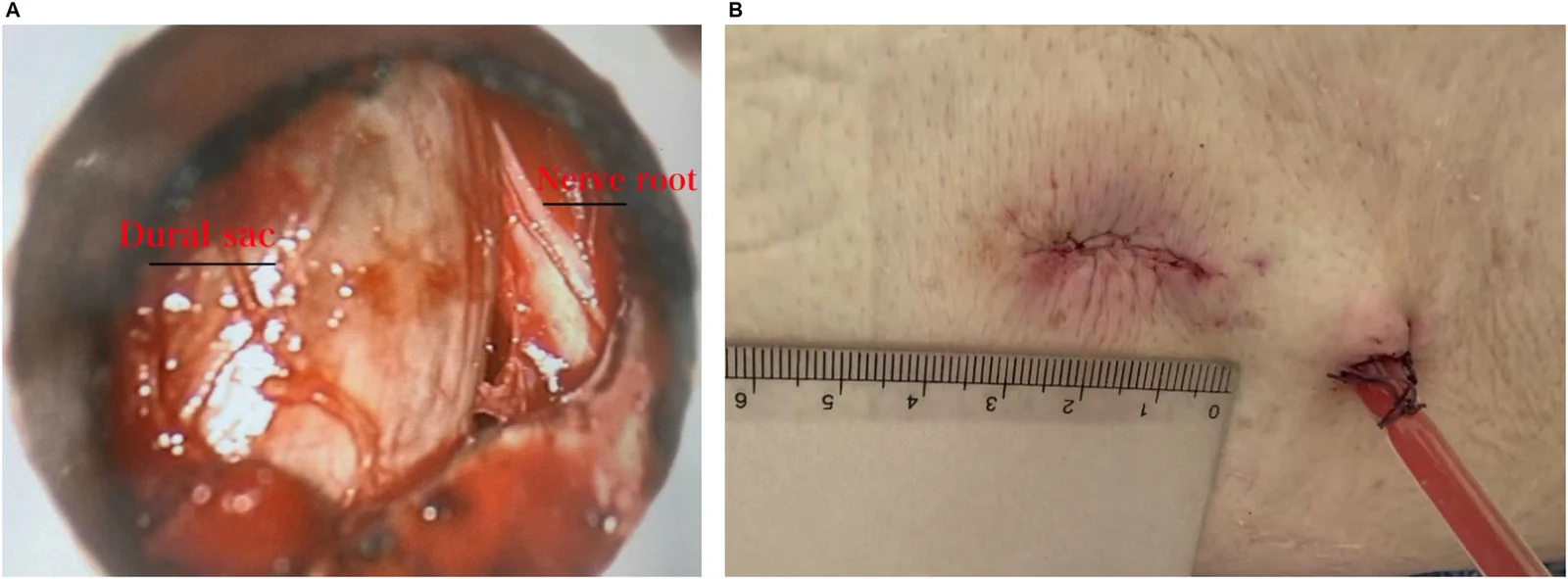
Unilateral microscopic channel-assisted decompression. **(A)** Microscopic visualization of the decompressed dural sac and nerve root. **(B)** Postoperative incision and drainage site. Alt text: Two-panel operative image showing microscopic neural decompression and the postoperative incision and drainage site.

### Clinical and radiographic assessment

Demographic characteristics, disease duration, decompression levels, operating time, estimated blood loss, postoperative hospital stay, and follow-up duration were extracted from the medical record. Disability was evaluated with the Oswestry Disability Index (ODI), expressed from 0 to 100, with higher scores indicating greater disability (18). The validity and clinical interpretation of ODI have been established in low-back-related disability (19), and standardized use is recommended in outcome research (20). Leg pain was evaluated using a 0–10 visual analog scale (VAS), with higher values indicating more severe pain (21). The clinical use and limitations of pain-rating scales have been reviewed previously (22). ODI and VAS improvement rates were calculated as (preoperative score−final-follow-up score)/preoperative score ×100%.

A common postoperative rehabilitation pathway was used. On postoperative day 2, patients underwent bilateral lower-extremity venous ultrasonography and began ambulation if no deep-vein thrombosis was detected. A lumbar brace was worn during the first postoperative month. Outpatient review was scheduled monthly for the first 3 months and every 3 months thereafter. Lumbar extensor exercises began after the first outpatient review. Walking duration and distance were increased gradually according to individual pain tolerance; therefore, actual walking exposure and adherence were not uniform or quantitatively recorded.

Standing whole-spine anteroposterior and lateral radiographs were obtained before surgery and at final follow-up using a GMM digital radiography system (GMM, Italy) equipped with a dedicated whole-spine grid; lateral images were used for sagittal measurements. For the anteroposterior view, participants stood with the back against the imaging stand, gaze forward, orbitomeatal line horizontal, palms facing forward, and arms relaxed at the sides. For the lateral view, the right side of the trunk was placed against the stand, the sagittal plane was parallel to the detector, feet were shoulder-width apart, knees were fully extended, and participants stood in a relaxed upright posture while looking forward. Both arms were advanced approximately 30° and supported with the forearms parallel to the floor and elbows flexed approximately 120°. All participants received standardized standing and breathing instructions before exposure. The x-ray tube moved vertically parallel to the detector, and multiple exposures were automatically stitched by the system. The same protocol was used at both visits. PT, pelvic incidence (PI), SS, LL, SVA, and TK were measured with Surgimap software (version 2.3.2.1, Nemaris Inc., New York, NY, USA) (23). PT was defined as the angle between the vertical and the line connecting the midpoint of the S1 endplate to the femoral head axis. PI was the angle between the line perpendicular to the S1 endplate and the line connecting its midpoint to the femoral head axis. SS was the angle between the S1 endplate and the horizontal. LL was measured from the superior endplate of L1 to the superior endplate of S1, TK from the superior endplate of T4 to the inferior endplate of T12, and SVA as the horizontal distance from the C7 plumb line to the posterosuperior corner of S1. The radiographic measurement approach is illustrated in Figure 3.

**Figure 3:**
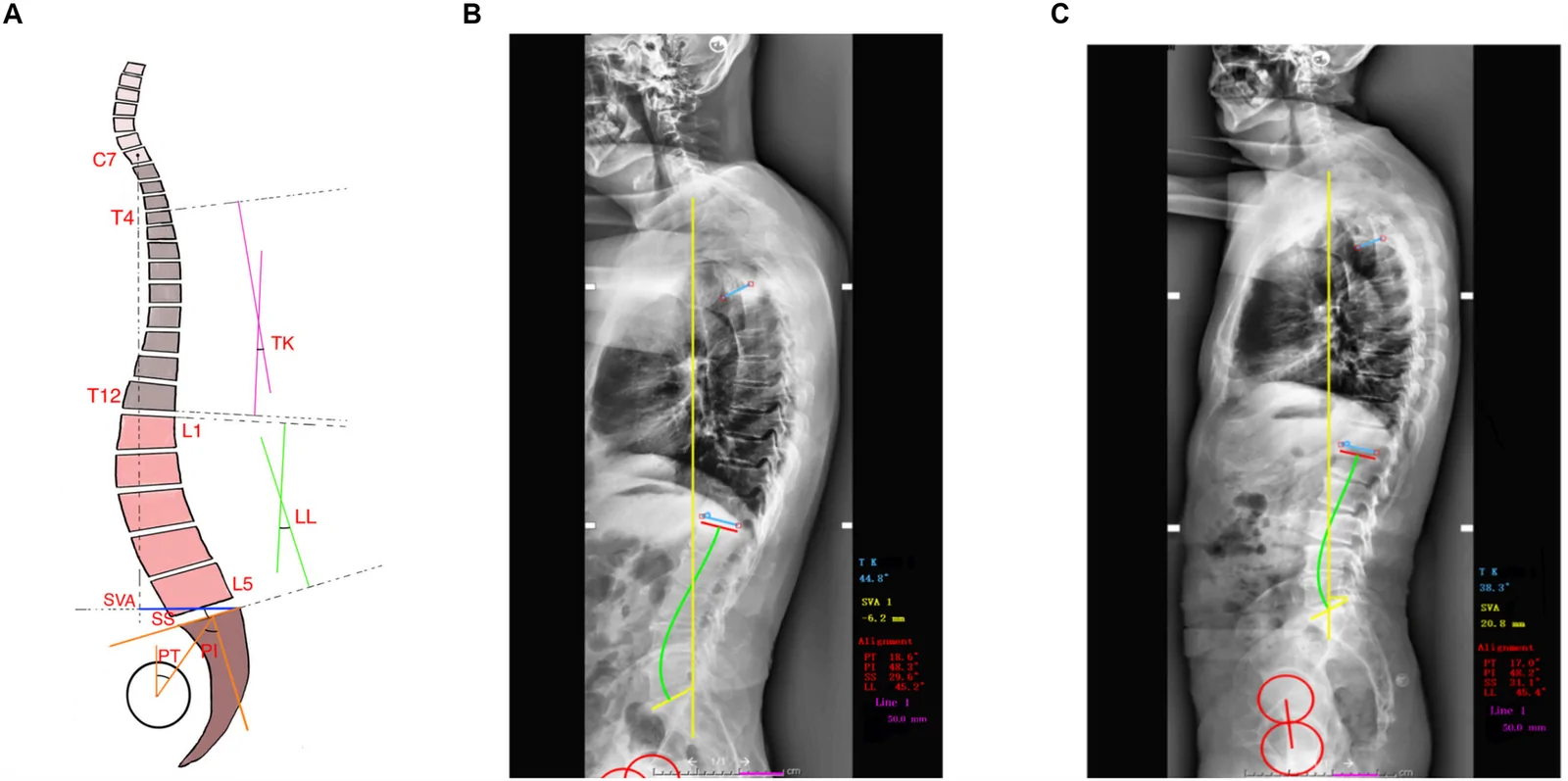
Radiographic assessment of spinopelvic sagittal parameters. **(A)** Schematic definitions of pelvic tilt (PT), pelvic incidence (PI), sacral slope (SS), lumbar lordosis (LL), sagittal vertical axis (SVA), and thoracic kyphosis (TK). **(B,C)** Representative standing whole-spine radiographs analyzed with Surgimap. Alt text: Three-panel radiographic figure showing definitions and representative measurements of spinopelvic sagittal parameters.

Two experienced spine physicians independently measured all preoperative and final-follow-up radiographs. Before measurement, images were anonymized, assigned study codes, stripped of patient and clinical information, and presented without labels identifying surgical level or preoperative/follow-up time point. The observers had no access to clinical outcomes. The first measurement session was conducted from 25 to 31 May 2026. After an interval exceeding one month, both observers repeated all measurements under the same blinded conditions. Observer 1 first-session values were used for the primary clinical and association analyses; paired first-session values were used for interobserver reliability, and the two sessions from each observer were used for intraobserver reliability.

### Statistical analysis

Continuous variables are reported as mean ± standard deviation (SD), and categorical variables as counts. Preoperative and final-follow-up measurements were compared using paired-samples t tests, with mean changes, 95% confidence intervals (CIs), and paired-sample Cohen dz reported. Change-score distributions were examined using Shapiro–Wilk tests; Wilcoxon signed-rank tests served as sensitivity analyses when appropriate.

For every radiographic parameter, change was calculated as the final-follow-up value minus the preoperative value (*Δ*=final follow-up−preoperative). Negative *Δ*PT and *Δ*SVA values therefore indicated postoperative decreases, while positive *Δ*SS, *Δ*LL, and *Δ*TK values indicated increases. PI was excluded from the change-correlation analysis because it is a morphologic parameter and did not change significantly.

Pearson correlation coefficients quantified the associations of changes in the five sagittal parameters with ODI and VAS improvement rates. Fisher 95% CIs and 10,000-resample bootstrap 95% CIs were calculated. The five sagittal changes were each tested against two clinical outcomes, yielding a family of 10 correlations. Holm's step-down procedure was selected to control the family-wise type I error while retaining more power than a single-step Bonferroni correction. A parsimonious multivariable linear regression then assessed whether *Δ*LL was independently associated with ODI improvement rate after adjustment for age, BMI, baseline ODI, and decompression extent (single vs. double level); HC3 heteroscedasticity-robust standard errors and variance inflation factors (VIFs) were reported. One- vs. two-level comparisons of clinical and radiographic changes were exploratory and used Welch t tests with Holm correction across eight outcomes. Separate *Δ*LL–ODI correlations were calculated by decompression extent, and their difference was assessed with Fisher's r-to-z test. In a separate exploratory analysis, Pearson correlations with Fisher 95% CIs examined whether follow-up duration in months was associated with *Δ*LL or *Δ*SVA; these two unadjusted analyses were interpreted descriptively. All tests were two-sided with *α*=0.05.

Interobserver and intraobserver reliability for each preoperative and final-follow-up sagittal parameter were quantified using two-way random-effects, absolute-agreement, single-measure intraclass correlation coefficients [ICC(A,1)] with 95% CIs (24, 25). Interobserver analyses compared the two physicians’ first-session measurements. Intraobserver analyses compared the two sessions separately for each physician. ICC values below 0.50 were interpreted as poor, 0.50–0.75 as moderate, 0.75–0.90 as good, and above 0.90 as excellent reliability (24).

Because this was a retrospective study, no formal *a priori* sample-size calculation was performed and all eligible patients were included. To describe the detectable correlation magnitude rather than to validate the observed results, a sensitivity calculation showed that *n* = 68 provided 80% power to detect a two-sided correlation of |r|≥0.33 at *α*=0.05. Effect estimates and 95% CIs were emphasized, and the sensitivity calculation was interpreted only as indicating that weaker secondary correlations and subgroup effects could have been missed. Analyses were performed using Python with SciPy 1.14 and statsmodels 0.14.

### Use of generative artificial intelligence

ChatGPT and Codex (GPT-5, OpenAI, San Francisco, CA, United States) were used solely to assist with English-language editing and manuscript formatting. The authors independently verified all scientific content, statistical results, interpretations, and references and take full responsibility for the final manuscript.

## Results

### Patient characteristics

Of 96 patients screened, 68 were included and 28 were excluded because postoperative whole-spine radiographs were incomplete or final VAS/ODI data could not be retrieved after loss to follow-up. Baseline variables for the excluded patients were not recoverable, precluding a formal included-vs.-excluded comparison. The analyzed cohort comprised 37 men and 31 women with a mean age of 64.57 ± 8.26 years. Thirty-four patients underwent single-level and 34 underwent double-level decompression. The single-level procedures comprised L2–3 in 1 patient, L4–5 in 16, and L5–S1 in 17. The double-level procedures comprised L3–4 plus L4–5 in 20 patients and L4–5 plus L5–S1 in 14. The mean follow-up was 8.65 ± 1.43 months (range, 6–12 months). Patient characteristics are summarized in Table 1.

**Table 1:** Patient characteristics (*n* = 68).

Characteristic	Value
Age, years	64.57 ± 8.26
Sex, male/female	37/31
BMI, kg/m^2^	26.26 ± 2.62
Symptom duration, months	42.54 ± 16.28
Single-/double-level decompression, n	34/34
Single-level: L2–3/L4–5/L5–S1, n	1/16/17
Double-level: L3–4 + L4–5/L4–5 + L5–S1, n	20/14
Operating time, min	155.66 ± 28.11
Estimated blood loss, mL	37.41 ± 12.22
Postoperative hospital stay, days	6.16 ± 1.14
Follow-up, months	8.65 ± 1.43 (range, 6–12)

Values are mean ± SD unless otherwise indicated. BMI, body mass index.

### Clinical and sagittal changes

ODI decreased by 41.37 percentage points (95% CI −45.86 to −36.88) and VAS decreased by 5.68 points (95% CI −5.98 to −5.38; both *p* < 0.001). The mean ODI and VAS improvement rates were 64.30 ± 14.27% and 79.37 ± 12.40%, respectively.

PT decreased by 2.84° and SVA by 3.77 cm, whereas SS increased by 9.99°, LL by 14.93°, and TK by 5.52° (all *p* < 0.001). PI changed by 1.21° and was not significantly different at follow-up (*p* = 0.437). Wilcoxon sensitivity analyses produced the same pattern of statistical significance (Table 2 and Figures 4, 5).

**Table 2:** Preoperative and final-follow-up clinical and sagittal measurements.

Variable	Preoperative	Final follow-up	Change, post−pre (95% CI)	p	Cohen dz
ODI	61.15 ± 18.55	19.77 ± 4.41	−41.37 (−45.86 to −36.88)	<0.001	−2.23
VAS	7.18 ± 1.20	1.50 ± 0.95	−5.68 (−5.98 to −5.38)	<0.001	−4.58
PT	15.30 ± 4.66	12.45 ± 4.36	−2.84 (−4.27 to −1.42)	<0.001	−0.48
PI	51.20 ± 8.93	52.41 ± 8.06	1.21 (−1.88 to 4.31)	0.437	0.09
SS	36.66 ± 7.71	46.64 ± 7.63	9.99 (7.41 to 12.56)	<0.001	0.94
LL	42.44 ± 13.81	57.37 ± 13.78	14.93 (10.70 to 19.16)	<0.001	0.85
SVA	−0.13 ± 1.20	−3.90 ± 1.23	−3.77 (−4.17 to −3.37)	<0.001	−2.28
TK	34.03 ± 7.64	39.54 ± 8.06	5.52 (2.75 to 8.28)	<0.001	0.48

Values are mean ± SD. *p* values are from paired-samples t tests.

ODI, Oswestry disability index; VAS, visual analog scale; PT, pelvic tilt; PI, pelvic incidence; SS, sacral slope; LL, lumbar lordosis; SVA, sagittal vertical axis; TK, thoracic kyphosis.

**Figure 4:**
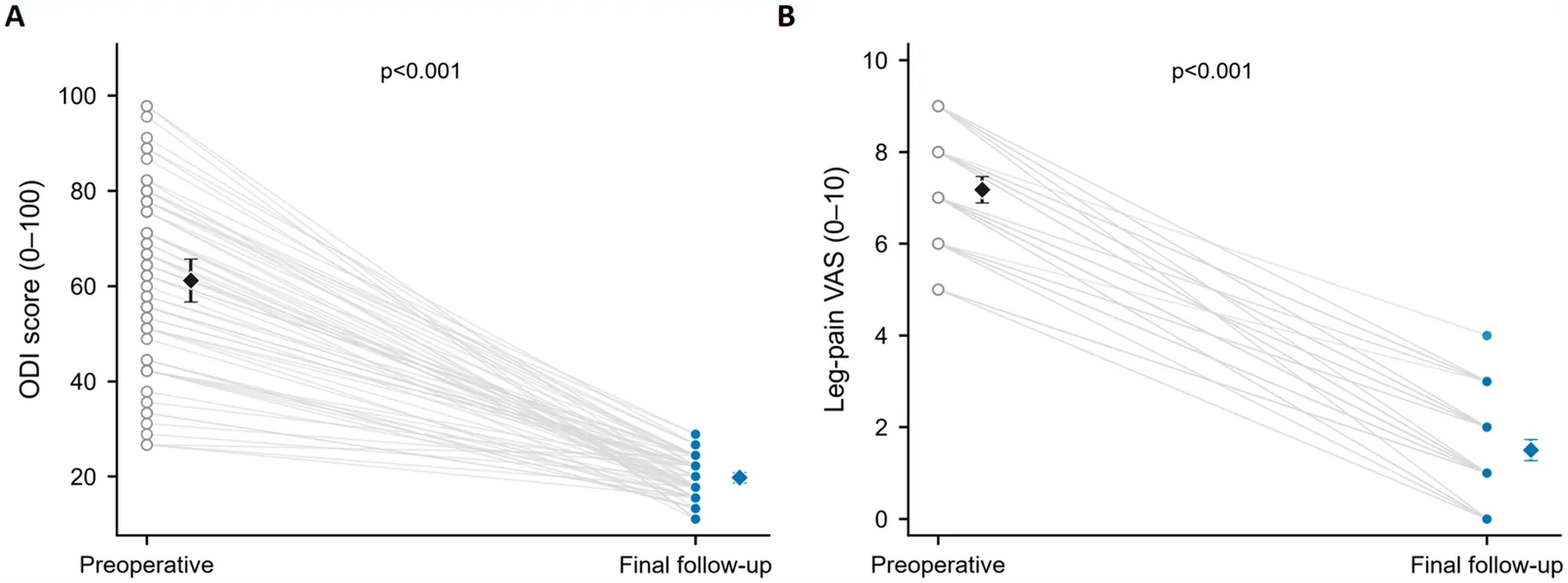
Individual clinical changes after unilateral microscopic decompression. Paired lines connect preoperative and final-follow-up observations for each patient (*n* = 68). Diamonds and error bars denote the mean and 95% confidence interval. *P* values are from paired-samples t tests. Alt text: Paired patient-level plots showing reductions in ODI and leg-pain VAS from preoperative assessment to final follow-up.

**Figure 5:**
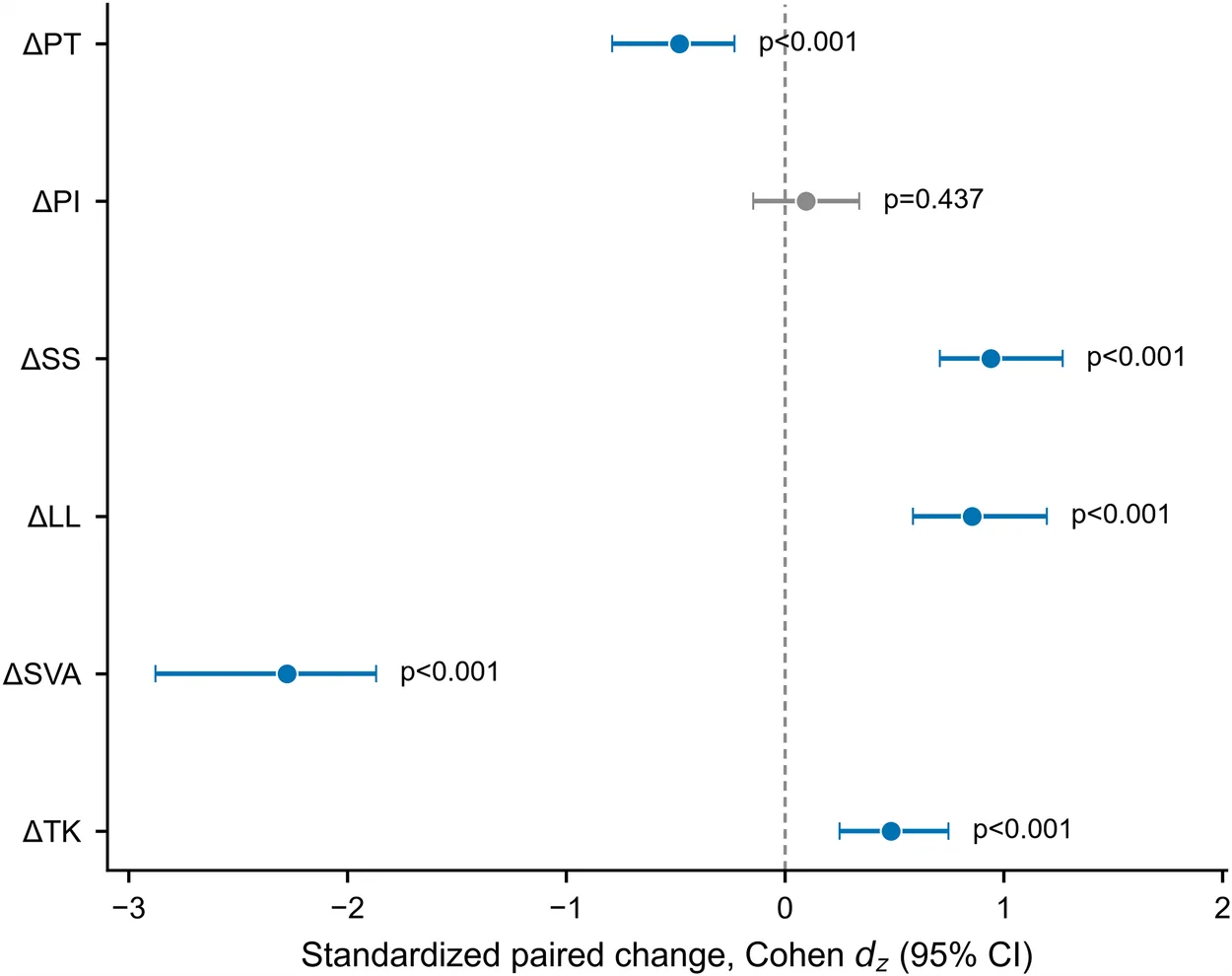
Standardized paired changes in standing sagittal parameters. Change was defined uniformly as the final-follow-up value minus the preoperative value. Points show paired-sample Cohen dz values and error bars show percentile-bootstrap 95% confidence intervals based on 5,000 resamples. Negative values indicate postoperative decreases and positive values indicate postoperative increases; PI did not change significantly. Alt text: Forest plot of standardized paired changes in PT, PI, SS, LL, SVA, and TK with bootstrap confidence intervals.

Follow-up duration was not associated with *Δ*LL (r = 0.093, 95% CI −0.148–0.325; *p* = 0.449) or *Δ*SVA (r = 0.045, 95% CI −0.195–0.280; *p* = 0.715) in the exploratory analyses.

### Interobserver reliability

Interobserver reliability was excellent for every preoperative and final-follow-up sagittal measurement, with ICC(A,1) point estimates ranging from 0.947 to 0.963. Intraobserver reliability was also excellent for both physicians, with point estimates ranging from 0.910 to 0.960. The corresponding 95% CIs are shown in Table 3.

**Table 3:** Interobserver and intraobserver reliability of sagittal parameter measurements.

Parameter/time point	Interobserver ICC (95% CI)	Observer 1 intraobserver ICC (95% CI)	Observer 2 intraobserver ICC (95% CI)	Interpretation
PT, preoperative	0.958 (0.924–0.974)	0.930 (0.885–0.958)	0.920 (0.848–0.956)	Excellent
PI, preoperative	0.952 (0.928–0.968)	0.950 (0.928–0.964)	0.940 (0.910–0.960)	Excellent
SS, preoperative	0.947 (0.913–0.968)	0.920 (0.870–0.950)	0.910 (0.853–0.944)	Excellent
LL, preoperative	0.961 (0.937–0.975)	0.910 (0.852–0.943)	0.930 (0.890–0.955)	Excellent
SVA, preoperative	0.955 (0.931–0.970)	0.940 (0.906–0.960)	0.950 (0.924–0.967)	Excellent
TK, preoperative	0.949 (0.919–0.968)	0.930 (0.892–0.954)	0.920 (0.878–0.947)	Excellent
PT, final follow-up	0.963 (0.934–0.978)	0.950 (0.914–0.969)	0.940 (0.902–0.961)	Excellent
PI, final follow-up	0.951 (0.924–0.969)	0.920 (0.876–0.947)	0.910 (0.853–0.943)	Excellent
SS, final follow-up	0.956 (0.931–0.970)	0.910 (0.839–0.950)	0.930 (0.883–0.957)	Excellent
LL, final follow-up	0.948 (0.923–0.964)	0.910 (0.861–0.941)	0.910 (0.863–0.939)	Excellent
SVA, final follow-up	0.960 (0.935–0.974)	0.960 (0.936–0.975)	0.960 (0.934–0.975)	Excellent
TK, final follow-up	0.953 (0.931–0.967)	0.930 (0.899–0.951)	0.920 (0.877–0.948)	Excellent

Values are two-way random-effects, absolute-agreement, single-measure intraclass correlation coefficients [ICC(A,1)] with 10,000-case bootstrap 95% confidence intervals (*n* = 68). Interobserver ICCs compare the physicians’ first sessions; intraobserver ICCs compare sessions at least one month apart. Interpretation is based on the point estimate. Abbreviations are defined in Table 2.

### Associations between sagittal parameter changes and clinical improvement

Greater LL increase was modestly associated with greater ODI improvement in the unadjusted analysis (r = 0.360, Fisher 95% CI 0.133–0.551; bootstrap 95% CI 0.197–0.526; *p* = 0.0026). This association remained significant after Holm correction across the 10 planned correlation tests (adjusted *p* = 0.026). *Δ*PT (r = −0.313, *p* = 0.009) and *Δ*SS (r = 0.279, *p* = 0.021) showed nominal unadjusted correlations with ODI improvement, but neither remained significant after multiplicity correction. *Δ*SVA and *Δ*TK were not correlated with ODI improvement. In the multivariable model adjusted for age, BMI, baseline ODI, and decompression extent, *Δ*LL was not independently associated with ODI improvement (B = −0.108 percentage points per degree, HC3 95% CI −0.276–0.060; *p* = 0.207). Baseline ODI was the only significant covariate (B = 0.673, HC3 95% CI 0.460–0.886; *p* < 0.001); all VIFs were <1.52 (Table 4).

**Table 4:** Multivariable linear regression for ODI improvement rate.

Term	B	HC3 SE	HC3 95% CI	p	VIF
Intercept	46.034	16.750	13.205 to 78.863	0.006	—
*Δ*LL, per degree	−0.108	0.086	−0.276 to 0.060	0.207	1.51
Age, per year	−0.106	0.191	−0.481 to 0.269	0.579	1.06
BMI, kg/m^2^	−0.591	0.482	−1.535 to 0.353	0.220	1.05
Baseline ODI, per point	0.673	0.109	0.460 to 0.886	<0.001	1.52
Double-level vs single-level	2.272	2.434	−2.498 to 7.043	0.351	1.02

Outcome: ODI improvement rate expressed as percentage points. Model R^2^ = 0.648; adjusted R^2^ = 0.620; *n* = 68. B, unstandardized coefficient; HC3, heteroscedasticity-consistent standard error; VIF, variance inflation factor.

None of the five sagittal changes was significantly correlated with VAS improvement. The primary LL association and complete correlation profile are presented in Figures 6, 7 and Table 5.

**Figure 6:**
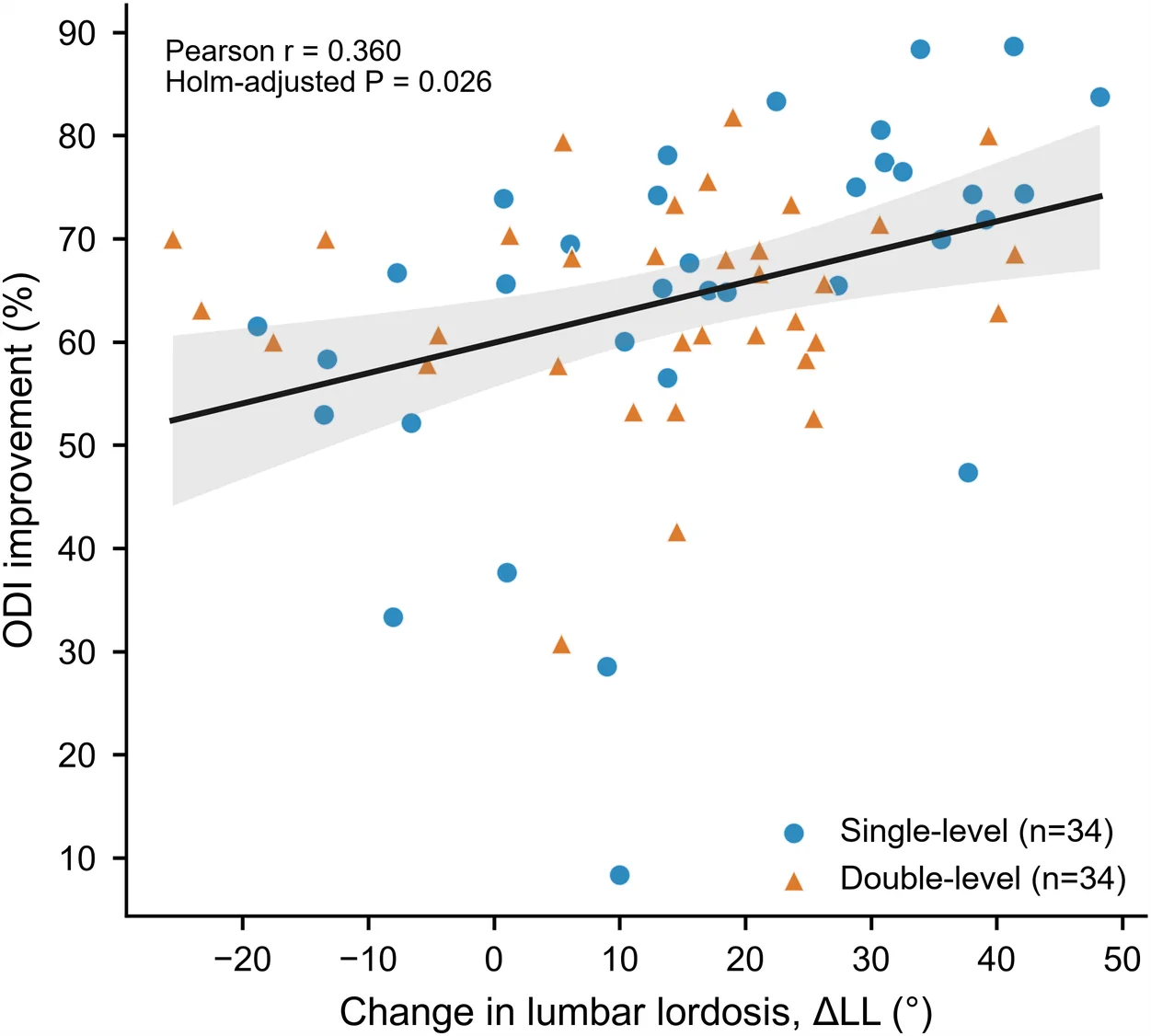
Association between change in lumbar lordosis and ODI improvement. Points represent individual patients and are coded by decompression extent. The black line shows the overall unadjusted linear fit and the shaded band its 95% confidence interval. The annotation reports the overall Pearson correlation coefficient and the Holm-adjusted *p* value across the 10 planned ODI/VAS correlation tests; the raw *p* value and Fisher 95% CI are reported in Table 5. Alt text: Scatter plot showing a modest positive unadjusted association between increased lumbar lordosis and ODI improvement, stratified by decompression extent.

**Figure 7:**
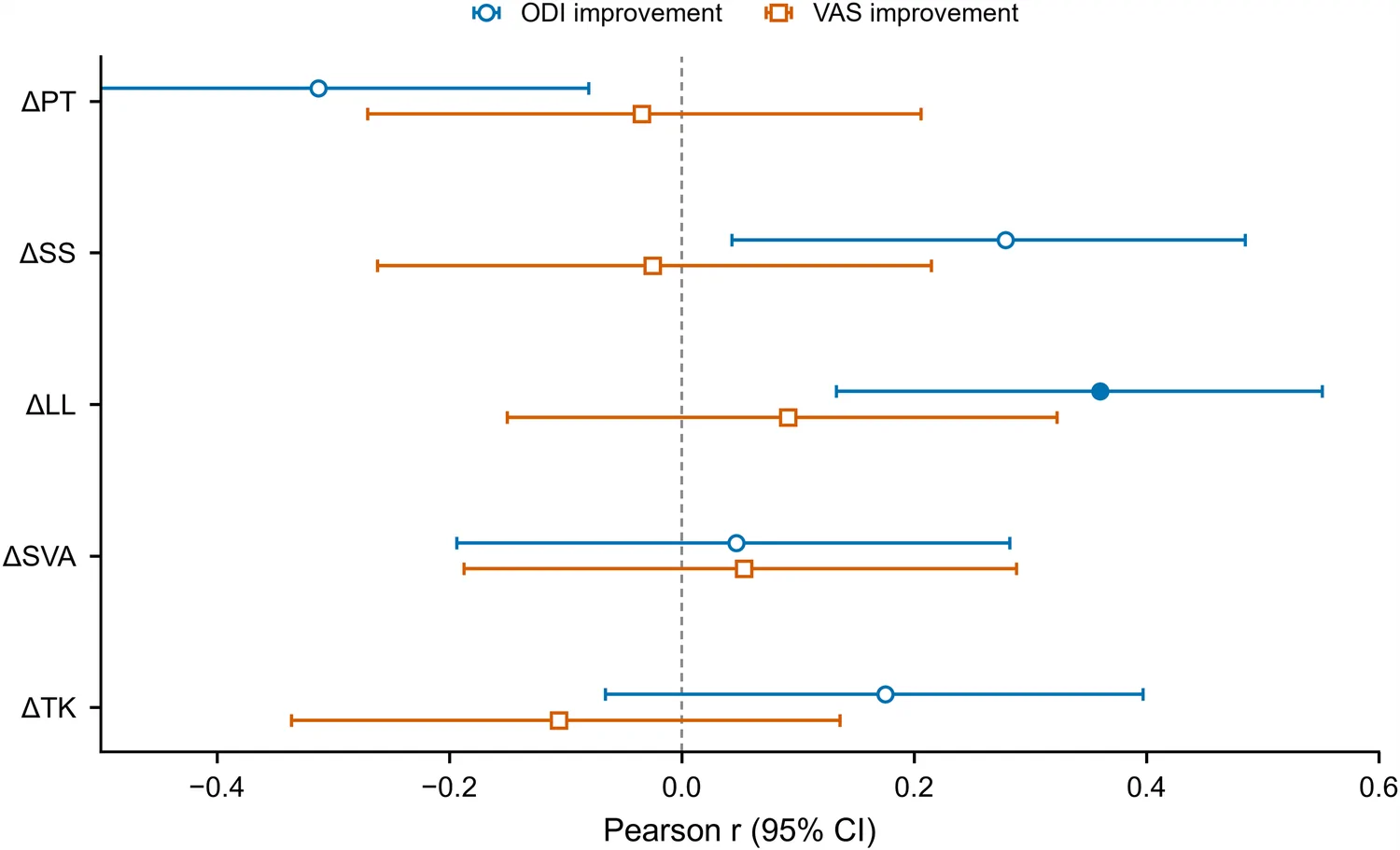
Correlations between changes in sagittal parameters and clinical improvement. Circles represent ODI improvement and squares represent VAS improvement. Points show Pearson correlation coefficients and error bars show Fisher 95% confidence intervals. Filled markers indicate Holm-adjusted *p* < 0.05 across the 10 planned tests. Alt text: Forest plot of correlation coefficients between sagittal parameter changes and ODI or VAS improvement rates.

**Table 5:** Correlations between changes in sagittal parameters and clinical improvement.

Sagittal parameter change	ODI improvement, r (95% CI)	ODI p raw/Holm	VAS improvement, r (95% CI)	VAS p raw/Holm
*Δ*PT (post−pre)	−0.313 (−0.513 to −0.080)	0.009/0.085	−0.034 (−0.271 to 0.206)	0.781/1.000
*Δ*SS (post−pre)	0.279 (0.043 to 0.485)	0.021/0.171	−0.025 (−0.262 to 0.215)	0.840/1.000
*Δ*LL (post−pre)	0.360 (0.133 to 0.551)	0.003/0.026	0.091 (−0.150 to 0.323)	0.458/1.000
*Δ*SVA (post−pre)	0.047 (−0.194 to 0.282)	0.705/1.000	0.053 (−0.187 to 0.288)	0.665/1.000
*Δ*TK (post−pre)	0.175 (−0.066 to 0.397)	0.153/1.000	−0.106 (−0.336 to 0.136)	0.390/1.000

Pearson correlations with Fisher 95% CIs are shown. Change was defined as the final-follow-up value minus the preoperative value. Holm correction was applied across all 10 ODI/VAS correlation tests. Abbreviations are defined in Table 2.

### Exploratory analysis by decompression extent

The single- and double-level groups each included 34 patients. No between-group difference in ODI improvement, VAS improvement, or changes in PT, PI, SS, LL, SVA, or TK remained significant after Holm correction across the eight comparisons (all adjusted *p* ≥ 0.335; Table 6). The unadjusted correlation between *Δ*LL and ODI improvement was r = 0.500 (95% CI 0.195–0.717; *p* = 0.003) in the single-level group and r = 0.120 (95% CI −0.227–0.440; *p* = 0.498) in the double-level group. The difference between correlations was not statistically significant (Fisher z = 1.69; *p* = 0.092), and no subgroup-specific causal interpretation was made.

**Table 6:** Exploratory comparison of single-level and double-level decompression.

Outcome/change	Single-level (*n* = 34)	Double-level (*n* = 34)	Raw p	Holm p
ODI improvement, %	64.62 ± 17.49	63.99 ± 10.37	0.857	1.000
VAS improvement, %	77.25 ± 14.06	81.49 ± 10.26	0.161	1.000
*Δ*PT,°	−3.78 ± 5.38	−1.90 ± 6.29	0.190	1.000
*Δ*PI,°	2.34 ± 12.80	0.08 ± 12.85	0.471	1.000
*Δ*SS,°	12.60 ± 10.55	7.37 ± 10.20	0.042	0.335
*Δ*LL,°	16.59 ± 18.36	13.27 ± 16.65	0.437	1.000
*Δ*SVA, cm	−3.62 ± 1.71	−3.92 ± 1.61	0.456	1.000
*Δ*TK,°	5.66 ± 11.83	5.37 ± 11.17	0.916	1.000

Values are mean ± SD. Change was defined as final follow-up minus preoperative value. Raw *p* values are from Welch t tests; Holm adjustment was applied across the eight exploratory comparisons.

## Discussion

During 6–12 months of follow-up, disability and leg pain decreased. PT and SVA also decreased, whereas SS, LL, and TK increased; PI remained unchanged. A greater increase in LL was modestly associated with ODI improvement in the unadjusted analysis and was the only correlation that remained significant after Holm correction. This association did not persist after adjustment for age, BMI, baseline ODI, and decompression extent. Radiographic and functional changes therefore occurred in parallel, but the data do not show that LL change caused or predicted recovery.

Interobserver ICC point estimates of 0.947–0.963 and intraobserver estimates of 0.910–0.960 indicate excellent reproducibility of landmark-based sagittal measurements. This reduces concern that the radiographic findings were driven by a single rater, although some intraobserver confidence limits crossed below 0.90 and measurement reliability cannot eliminate variability from patient posture or image acquisition. Full blinding of both physicians to patient identity, clinical outcomes, surgical level, and imaging time point further limited measurement-expectation bias.

ODI and VAS fell substantially during follow-up, in line with previous reports of minimally invasive decompression for symptomatic LSS (26). Short-term improvement has also been reported after unilateral laminotomy with bilateral decompression (27). The unilateral microscopic approach decompresses both sides while limiting disruption of posterior stabilizing structures (28); comparisons with open laminectomy support this rationale (29). Postoperative activity and physical therapy may affect longer-term recovery (30). Early mobilization, bracing, scheduled review, and the start of lumbar extensor exercises followed a common protocol in our cohort, but walking exposure varied with pain tolerance and was not quantified. Without a nonoperative or alternative-surgery comparison group, we cannot determine how much of the observed improvement was specific to this technique or whether it is superior to other approaches.

Reports of sagittal change after decompression alone are not uniform. Fujii et al. observed increases in LL and TK with reductions in SVA and PT (10); Madkouri et al. found increased LL and decreased SVA at one year (11); and Bouknaitir et al. reported improvement in SVA and PI–LL mismatch (12). Sagittal improvement has also been described after microendoscopic laminotomy (13). A systematic review found marked heterogeneity across laminectomy studies (31), which may reflect differences in baseline alignment, positioning, symptom severity, surgical technique, and follow-up.

No fusion or corrective instrumentation was used, so these changes should not be interpreted as structural reconstruction. In a different population, Gulec et al. observed a small improvement in segmental kyphosis without deterioration of global sagittal balance after unilateral biportal endoscopic decompression for stable adjacent-segment disease without extension of fusion (32). Their cohort had undergone previous lumbar fusion and is not directly comparable with patients treated for primary LSS. The study nonetheless shows that sagittal alignment can remain stable after decompression in carefully selected segments. A reduction in pain-avoidant flexion may partly explain our findings. Patients with LSS often lean forward to reduce symptoms and improve walking tolerance (33), and lumbar degenerative disease can produce compensatory changes across the pelvis and spine (34, 35). Once neural compression and pain improve, some patients may stand more upright, with reciprocal pelvic or thoracic changes similar to those reported after surgery for other lumbar degenerative conditions (36, 37). We did not measure this mechanism directly. All included patients had <3 mm motion on preoperative flexion-extension radiographs, which makes instability-driven realignment less likely.

The unadjusted *Δ*LL–ODI correlation was modest (r = 0.360). After adjustment, the coefficient changed direction and was no longer statistically significant, while baseline ODI remained strongly associated with percentage improvement. The improvement rate contains the baseline score in its denominator, so part of this attenuation may arise from baseline dependence as well as clinical confounding. The result is a bivariate association within this cohort, not evidence of independent prediction. ODI recovery may also depend on neural decompression, initial symptom severity, physical condition, psychosocial factors, and rehabilitation.

Although the point estimates differed between the single- and double-level groups, the formal comparison between correlations was not statistically significant. With only 34 patients in each subgroup, however, the confidence intervals were wide and the power to detect effect modification was limited. A true difference by decompression extent therefore cannot be excluded, and the observed trend should be regarded as hypothesis-generating rather than evidence of a subgroup effect.

Published radiographic–clinical findings are also mixed. Some decompression cohorts found no significant association, whereas others reported weak relationships involving SVA or postoperative alignment (10, 12, 14, 38). Our results likewise do not show that an increase in LL produced the functional recovery.

Sagittal changes did not correlate with VAS improvement. Leg pain fell rapidly in most patients, leaving a narrow range of postoperative VAS scores and a possible floor effect. Pain relief also follows neural decompression more directly than changes in standing posture, which may take longer. ODI includes walking, standing, lifting, sitting, and other daily activities, so its relationship with alignment need not match that of residual leg pain.

This study has several limitations. Its retrospective, single-center design restricts generalizability and precludes causal inference. Twenty-eight of 96 screened patients were excluded because postoperative radiographs or follow-up ODI/VAS data were unavailable. Their baseline records could not be recovered, so we could not assess selection bias directly. The sensitivity calculation described the correlation magnitude detectable with this sample but did not replace *a priori* planning; weak secondary or subgroup effects may have been missed, and residual confounding remains possible. Follow-up lasted 6–12 months. Follow-up duration was unrelated to *Δ*LL or *Δ*SVA in the exploratory analyses, but this narrow interval cannot show whether sagittal changes persist, progress, or regress later. Standardized positioning and the ICC results support measurement reliability, although posture may still vary with pain and fatigue. Early rehabilitation followed a common protocol, but walking exposure and adherence were individualized and not recorded quantitatively. The findings apply to patients with <3 mm motion on flexion-extension radiographs and should not be extrapolated to patients who require fusion or deformity correction. The possibility that excluded patients differed from the analyzed cohort with respect to spinopelvic radiographic features or clinical outcomes cannot be excluded.

## Conclusions

During 6–12 months of follow-up after unilateral microscopic channel-assisted decompression, ODI and leg-pain VAS decreased and several standing spinopelvic parameters changed. A greater increase in LL was modestly associated with greater ODI improvement in the unadjusted analysis, but not after adjustment for age, BMI, baseline ODI, and decompression extent. The sagittal changes may represent postural adaptation rather than structural correction, but this observational study cannot establish causality. Prospective multicenter studies with longer follow-up and measured rehabilitation exposure are needed to clarify how alignment and functional recovery evolve over time.

## Data Availability

The raw data supporting the conclusions of this article will be made available by the authors, without undue reservation.
